# Discussion on operation: To compare the curative effect of PMT and CDT in the treatment of middle and high risk stratified APE and the clinical application value of serum BNP, TnI and plasma DFR levelse

**DOI:** 10.3389/fsurg.2023.1091823

**Published:** 2023-02-01

**Authors:** Qinglong Guan, Chenglong Liu, Wei Li, Xiaofei Wang, Ruiyuan Gu, Ruihua Wang, Gang Li, Shuai Liu

**Affiliations:** ^1^ Department of Vascular Surgery, The Second Affiliated Hospital of Shandong First Medical University, Taian, China; ^2^ Department of Radiology, The Second Affiliated Hospital of Shandong First Medical University, Taian, China; ^3^ Department of Vascular Surgery, The Second Hospital of Yinzhou District, Ningbo, China; ^4^ Department of Medical Laboratory, The Second Affiliated Hospital of Shandong First Medical University, Taian, China; ^5^ Department of Vascular Surgery, The Ninth People’s Hospital Affiliated to the Medical College of Shanghai Jiaotong University, Shanghai, China; ^6^ Department of Scientific Research, Shandong First Medical University, Jinan, China

**Keywords:** aute pulmonary embolism, percutaneous mechanical thrombolysis, catheter directed thrombolysis, B-type brain natriuretic peptide, troponin I, D-dimer/fibrinogen ratio

## Abstract

**Objective:**

To compare the efficacy of Percutaneous mechanical thrombectomy (PMT) and Catheter directed thrombolysis (CDT) in the treatment of patients with moderate and high-risk ape and explore the clinical application value of biomarkers in the treatment of moderate and high-risk ape.

**Method:**

A total of 84 patients with ape were selected from the Department of vascular surgery of the Second Affiliated Hospital of Shandong First Medical University and the Department of vascular surgery of Shanghai Ninth People's Hospital Affiliated with Shanghai Jiao Tong University School of Medicine. According to the relevant guidelines, they were divided into high-risk and medium-risk groups, including PMT groups (35 cases) and CDT groups (49 cases). To detect the changes of serum B-type brain natriuretic peptide (BNP),Troponin I (TnI) and plasma D-dimer/fibrinogen ratio (DFR) levels in different risk stratification before and after PMT and CDT, the correlation and diagnostic value of each index, and compare the thrombus clearance rate, pulmonary artery pressure, average dosage of urokinase, effective thrombolytic time, average hospitalization time and complications of PMT and CDT.

**Result:**

Under different treatment methods and risk stratification, there was no statistically significant difference in the clinical data of patients at general baseline;The preoperative BNP, TnI and DFR levels of PMT and CDT in the middle and high risk stratification were significantly lower than those in the other groups (*P *< 0.005),Compared with the CDT group, PMT has significantly better therapeutic effect on ape than the CDT group in terms of thrombus clearance rate, pulmonary artery pressure, average dosage of urokinase, effective thrombolytic time and average hospitalization time (*P *< 0.05),meanwhile,there was no significant difference in postoperative complications between the two groups (*P *< 0.05). After half a year of follow-up, the levels of BNP, TnI and DFR in the cured group were significantly lower than those in the effective group and the ineffective group. The areas under the curve of serum BNP, TnI and plasma DFR were 0.91, 0.87 and 0.93 and the area under the curve DFR has higher diagnostic efficiency than BNP and TnI, while the sensitivity and specificity of TnI are significantly higher than BNP and DFR.

**Conclusion:**

Serum BNP, TnI and plasma DFR levels can reflect the risk stratification and better clinical diagnostic value of ape,PMT and CDT are used to treat high-risk ape. For hospitals with medical conditions, PMT is more worthy of clinical recommendation.

## Introduction

Acute pulmonary embolism (APE) is a disorder of pulmonary circulation caused by embolus in the venous system that block the trunk of the pulmonary artery or its branches (1). APE has atypical clinical symptoms due to different severity of the disease, and is prone to misdiagnosis and missed diagnosis. if the treatment indication is incorrect, the mortality rate of all causes in one week is 1.9%–2.9%, and that of all-cause cases within one month is 4.9%–6.6% (2). Clinically, according to 2015 Esc/Ers Guidelines for the Diagnosis and Treatment of Pulmonary Hypertension (3), the hemodynamic stability was divided into low risk stratification, medium risk stratification and high risk stratification. Anticoagulant therapy only for low risk stratified APE, and for medium and high risk stratified APE, there are mainly therapy such as Percutaneous mechanical thrombolysis, (PMT) (4), Catheter directed thrombolysis (CDT) and peripheral venous thrombolysis. Biological indexes such as B-type brain natriuretic peptide (BNP), Troponin I (TnI) (5) and D-dimer/fibrinogen ratio (DFR) are often used as indicators for the diagnosis and treatment of acute cardiopulmonary diseases. Among them, BNP (6), as a common diagnostic index of APE, is a reliable index to evaluate the short-term prognosis of pulmonary embolism. APE can cause pathophysiological changes such as acute pulmonary artery pressure increase, hemodynamic disturbance and gas exchange disturbance, and further damage cardiomyocytes, affect myocardial metabolism and reduce cardiac function. Therefore, plasma TnI is usually used as a common diagnostic index in clinical APE, which can evaluate the severity of cardiopulmonary function damage. D-dimer/fibrinogen ratio (7) can be used as an active index of thrombosis. At present, it is widely used in diseases such as venous thrombosis and ischemic stroke, and its sensitivity is higher than that of simple D-dimer.

The purpose of this study is to explore the efficacy analysis of PMT and CDT before and after the treatment of high-risk APE, the changes of serum BNP, TnI and DFR levels and clinical application value, so as to provide help for the diagnosis and treatment of clinical APE.

## Materials and methods

### Research subjects

A total of 84 patients with APE were selected from the Second Affiliated Hospital of Shandong First Medical University and the Shanghai Ninth People’s Hospital Affiliated with Shanghai Jiao Tong University School of Medicine from June 2019 to June 2022. Diagnostic criteria were referred to the Treatment and Prevention of Heparin-Induced Thrombocytopenia: Antithrombotic Therapy and Prevention of Thrombosis (8) and the 2019 ESC Guidelines for the diagnosis and management of acute pulmonary embolism developed in collaboration with the European Respiratory Society (ERS) (9).

Diagnostic criteria include: echocardiography/large right ventricle and/right atrium, leftward shift of septum, proximal pulmonary artery dilatation, increased tricuspid regurgitation velocity, etc.; pulmonary CTA/manifestation of intrapulmonary filling defect, Blood Gas Analysis (BGA)/Hypoxaemia, Hypocapnia, Electrocardiographic (ECG)/inversion of T waves in leads V1–V4, a QR pattern in V1, a S1Q3T3 pattern, and incomplete or complete right bundle branch block. Risk stratification was based on hemodynamic stability including hypotension, shock, pulmonary embolism severity index, right ventricular insufficiency, dyspnoea, chest pain, haemoptysis. According to hemodynamic stability or instability, Inclusion criteria: High-risk group: hemodynamic instability, mainly characterized by shock or persistent hypotension (systolic blood pressure <90 mmHg or systolic blood pressure drop ≥40 mmHg persistent 15 min); Medium-risk group: echocardiography or CT indicates right ventricular insufficiency and/or elevated levels of cardiac biomarkers troponin and B-type brain natriuretic peptide; Exclusion criteria: Low-risk group: hemodynamic stability, no right ventricular dysfunction and normal levels of cardiac biomarkers.

### Methods

In both groups, patients were given 0.1 ml/kg subcutaneous injection of low molecular heparin calcium anticoagulation (Tianjin Hongri Pharmaceutical Co., Ltd., State Pharmacopoeia H2020470, specification: 0.6 ml/branch) immediately after the diagnosis of APE. In both groups, the right inguinal area was disinfected (right side was the main one), the right femoral vein was punctured with the Seldinger method under local anesthesia and a vascular sheath was placed, and an inferior vena cava filter (Denali, Bard, United States) was placed under the opening to prevent thrombus dislodgement.

PMT: Routine puncture of the right femoral vein or puncture of the contralateral femoral vein according to the results of ultrasound. An 8F vascular sheath was placed through the femoral vein, and a 5F pigtail catheter was led through the sheath with a mudskipper guidewire to the left and right pulmonary arteries successively for imaging, and to determine the size, location and degree of embolization of the thrombus. Afterwards, a stiffened guidewire was placed distal to the embolization site. Then the AngioJet embolization catheter was fed along the guidewire. Adjust the spray mode, spray 200,000 IU of urokinase with 100 ml of saline solution on the thrombus and wait for 15 min. Adjust the extraction mode and repeatedly aspirate the thrombus for <5–10 s each time, paying attention to the presence or absence of vagal reflexes during the period. After completion of thrombus extraction, the pigtail catheter was reintroduced and pulmonary arteriography was performed again to understand the effect of thrombus aspiration.

CDT treatment: 5F catheter sheath was placed through femoral vein, 5F pigtail catheter was inserted for pulmonary arteriography to clarify the site, size and morphology of thrombus, the catheter was disposed into the involved thrombus site, the head end of the catheter was turned for thrombus fragmentation, and subsequently the thrombolytic catheter was placed in the pulmonary artery for 10 min to infuse about 600,000 IU of urokinase (loading dose 4,000 IU/kg) for thrombolysis, and the catheter was left in place for continuous Urokinase was pumped (for 48 h) at a dose of about 2,000 IU/kg.h continuously. During this period, the patient's coagulation analysis was monitored, and catheterization was performed every 24–48 h to observe the effect of thrombolysis.

### Observation indicators and efficacy evaluation criteria

Laboratory examination: 4 ml of fasting venous blood was collected from patients, and the plasma was separated by centrifugation at 3,000 r/min for 10 min, and the supernatant was stored in a refrigerator at −80°C to be measured. The DFR was calculated based on the test results. BNP, TnI, and DFR levels were compared between the two groups, and the above indexes were operated strictly according to the kit instructions. All patients were followed up until six months after discharge, and were divided into cure group, effective group and invalid group according to the efficacy evaluation criteria, and the imaging evaluation criteria: cure group/patients with complete remission of symptoms, complete restoration of vascular blood flow and no complications; effective group/patients with partial remission of symptoms, partial restoration of blood flow and a small amount of complications; invalid group/patients with no remission of symptoms and obvious intravascular emboli.

### Statistical methods

Data were analyzed using SPSS22.0 statistical software, and the count data conforming to normal distribution were expressed as ($\bar{X}\pm S$), and the sample *t*-test was used for comparison between groups, and one-way ANOVA was used for comparison between multiple groups; the count data were expressed as number of cases, and *χ*^2^ test was used for comparison, and Spearman rank correlation was applied for correlation analysis; the area under the ROC curve analysis was used to evaluate the prognostic prediction of each index, and *P* < 0.05 was considered a statistically significant difference.

## Results

### Comparison of the basic data of each group

Under two different treatment methods and different risk stratification, the patient's age, gender (M/F), DVT history, trauma history, cardiovascular disease, and bed rest were not statistically significant compared with the high-risk risk group (*P* > 0.05, Table 1).

**Table 1:** General clinical baseline data.

Clinical Data	PMT		CDT		*χ* ^2^	*P*-Value
	Medium-risk group (*n* = 26)	High-risk group (*n* = 9)	Medium-risk group (*n* = 37)	High-risk group (*n* = 12)		
Age	59.30 ± 12.35	59.44 ± 7.61	62.13 ± 10.59	65.50 ± 9.80	1.23	0.27
Gender (M/F)	16/10	5/4	22/15	7/5	0.06	0.79
DVT-Medical history (With/without)	19/7	6/3	23/14	9/3	0.16	0.68
Trauma history (<1Month) (With/without)	7/19	4/5	11/26	3/9	0.17	0.67
History of surgery (With/without)	4/22	2/7	10/27	3/9	0.02	0.88
Cardiovascular disease (with/without)	16/18	7/2	28/9	9/3	0.31	0.57
Bed (>14 days) (with/no)	5/21	2/7	6/31	4/8	1.21	0.27

### Comparison of serum BNP, TnI and DFR levels between two groups of APE patients in different risk stratification before and after operation

Compared the levels of serum BNP, TnI and plasma DFR in patients with different APE risk stratification before and after PMT or CDT treatment, the levels of serum BNP, TnI and DFR after operation were significantly lower than those before treatment, with statistical significance (*P* < 0.05). In APE treated with PMT, the *F* values of BNP, TnI and DFR before and after the operation at the intermediate risk level were 24.1, 12.81 and 4.64 respectively; In high-risk risk stratification, the *F* values of BNP, TnI and DFR before and after operation were 6.45, 6.10 and 6.56 respectively. In APE treated with CDT, the *F* values of BNP, TnI and DFR before and after operation in the middle risk stratification were 6.71, 4.77 and 4.05 respectively; *F* values of BNP, TnI and DFR in high-risk risk stratification before and after operation are 12.15, 5.62 and 5.76 respectively, as shown in Table 2.

**Table 2:** Comparison of preoperative and postoperative serum BNP, TnI and DFR levels between two groups of APE patients.

Group	Index	PMT		CDT	
		Medium-risk group	High-risk group	Medium-risk group	High-risk group
Preoperative	BNP	833.36 ± 372.18^a^	1992.92 ± 536.66^aa^	794.59 ± 263.01^d^	2073.24 ± 435.89^dd^
	TnI	3.48 ± 1.41^b^	5.69 ± 1.05^bb^	3.74 ± 1.26^e^	7.24 ± 2.13^ee^
	DFR	4.14 ± 1.36^c^	5.47 ± 1.08^cc^	4.58 ± 1.24^f^	7.71 ± 2.01^ff^
Postoperative	BNP	165.06 ± 96.52^a^	378.62 ± 140.77^aa^	249.49 ± 165.60^d^	811.42 ± 146.93^dd^
	TnI	0.84 ± 0.62^b^	1.19 ± 0.56^bb^	1.85 ± 0.81^e^	4.45 ± 1.09^ee^
	DFR	1.12 ± 0.78^c^	2.77 ± 1.09^cc^	1.80 ± 0.97^f^	3.58 ± 1.20^ff^

PMT: a, b, c for BNP, TnI, and DFR in the intermediate risk stratum, aa, bb, cc for BNP, TnI, and DFR in the high risk stratum, CDT: d, e, f for BNP, TnI, and DFR in the intermediate risk stratum, dd, ee, ff for BNP, TnI, and DFR in the high risk stratum.

### Comparison of surgical efficacy related indicators between the two groups

In APE with medium and high risk, compared with CDT group, PMT group had significantly better treatment of APE than CDT group in terms of thrombus clearance rate, pulmonary artery pressure, average dosage of urokinase, effective thrombolytic time and average hospital stay during treatment (*P* < 0.05). Both PMT and CDT groups had related complications during and after the operation. In PMT group, chest tightness, decreased blood pressure, blood leakage around the vascular sheath and postoperative hemoglobinuria were easy to occur during the operation. In CDT group, the above symptoms also occurred when urokinase was used. The incidence of complications in the two groups was similar, but the difference was not statistically significant (*P* = 0.83), as shown in Table 3.

**Table 3:** Comparison of indicators related to surgical efficacy in the two patient groups.

Group	PMT		CDT		*χ* ^2^	*P*-Value
	Medium-risk group	High-risk group	Medium-risk group	High-risk group		
Thrombotomy rate (%)	83.32 ± 10.53	85.91 ± 6.35	64.57 ± 12.65	63.83 ± 12.85	28.54	0.04
PAP (mmHg)	29.96 ± 6.09	29.22 ± 7.67	38.91 ± 8.79	36.41 ± 9.63	34.64	0.04
Effective thrombolysis time (H)	2.43 ± 3.21	3.73 ± 1.22	4.24 ± 0.80	4.35 ± 0.69	79.71	0.04
Urokinase usage (Ten thousand units U)	31.30 ± 10.33	55.55 ± 11.30	177.16 ± 63.22	234.16 ± 52.30	56.78	<0.01
Average stay (D)	3.19 ± 0.42	3.38 ± 0.65	5.34 ± 1.02	6.83 ± 0.83	61.42	<0.01
Complications (Case%)	7.19 ± 3.65	3.66 ± 1.65	4.59 ± 2.45	10.37 ± 3.75	71.0	0.83

### Comparison of serum BNP, TnI and DFR levels in patients with APE with different prognosis after two kinds of surgical treatment

**Figure 1:**
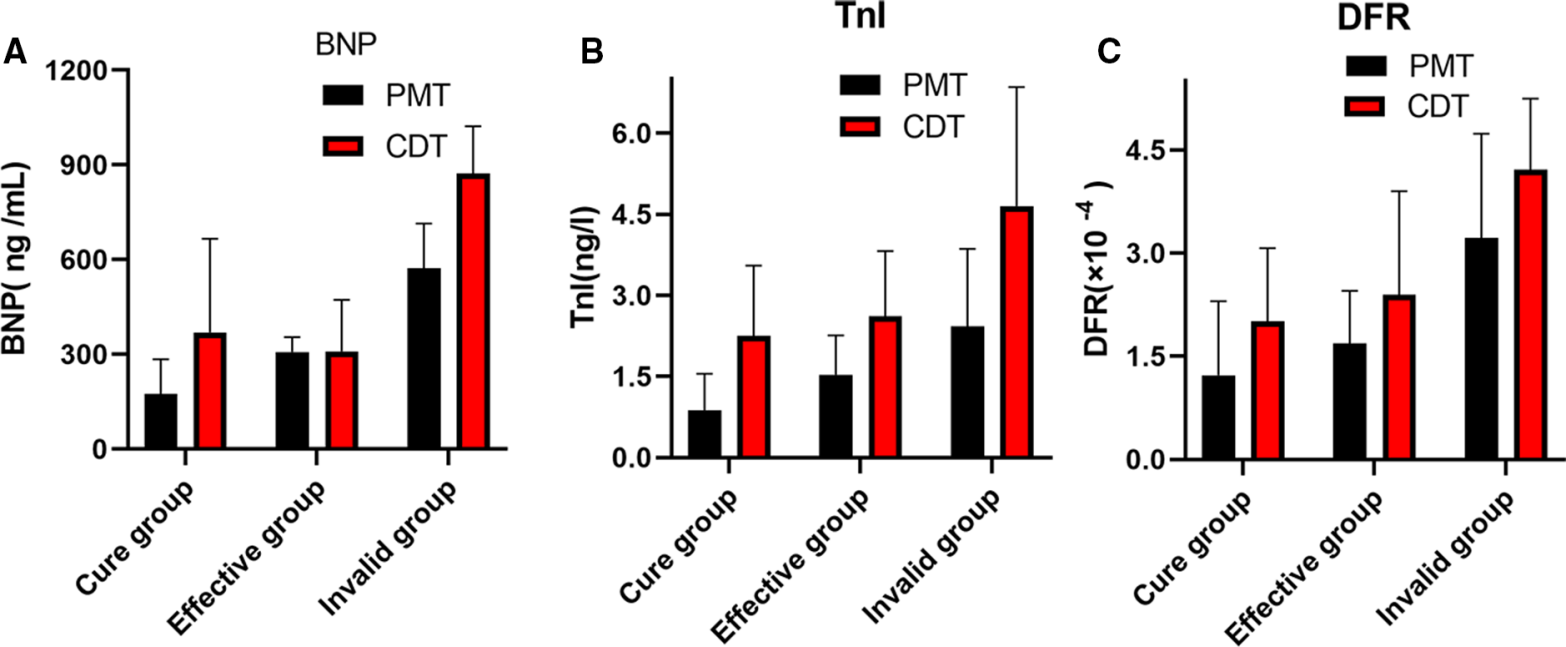
(**A–C**) Comparison of serum BNP,TnI and DFR levels in patients with APE after PMT and CDT treatment.

The patients with APE were followed up for half a year after PMT and CDT treatment (Figures 1A–C). The levels of serum BNP, TnI and plasma DFR in the cured group were significantly lower than those in the effective and ineffective groups (*P* < 0.05); The serum BNP, TnI and DFR levels in the effective group were significantly lower than those in the ineffective group (*P* < 0.05).

### Correlation analysis of serum BNP, TnI and DFR levels in patients before and after operation

**Figure 2:**
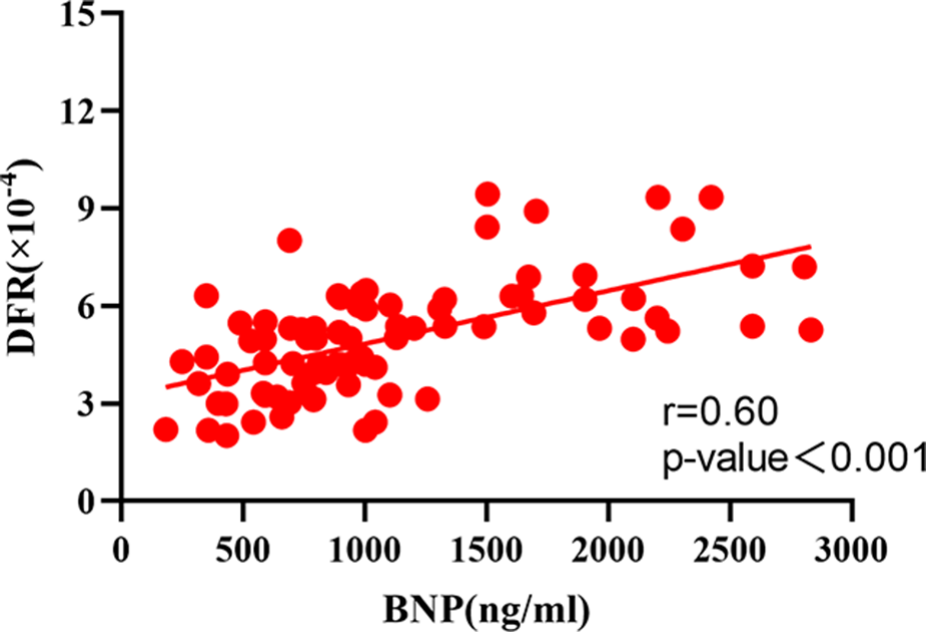
Correlation between serum BNP and DFR before operation.

**Figure 3:**
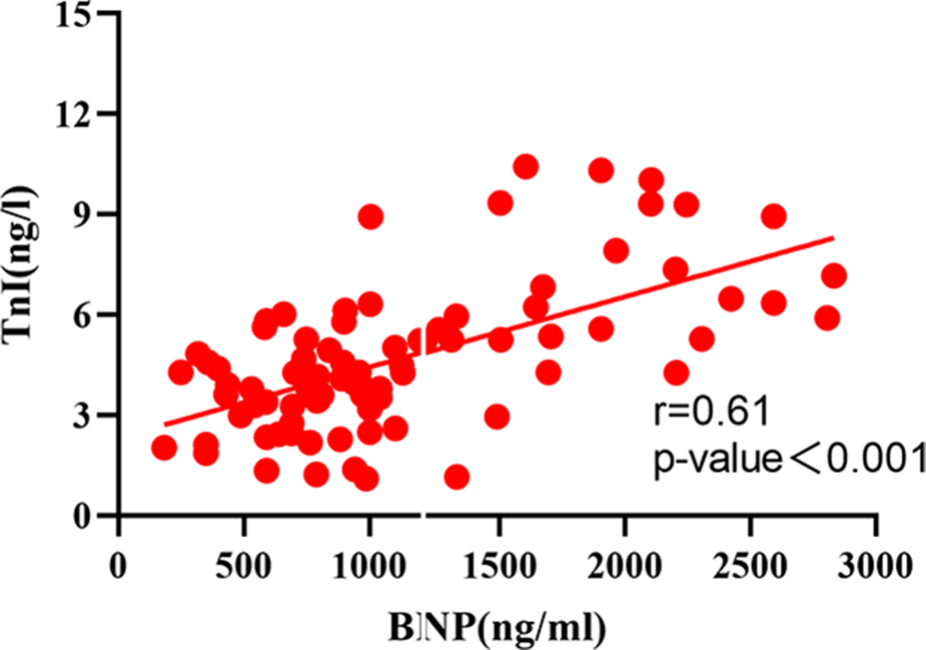
Correlation between serum BNP and TnI before operation.

**Figure 4:**
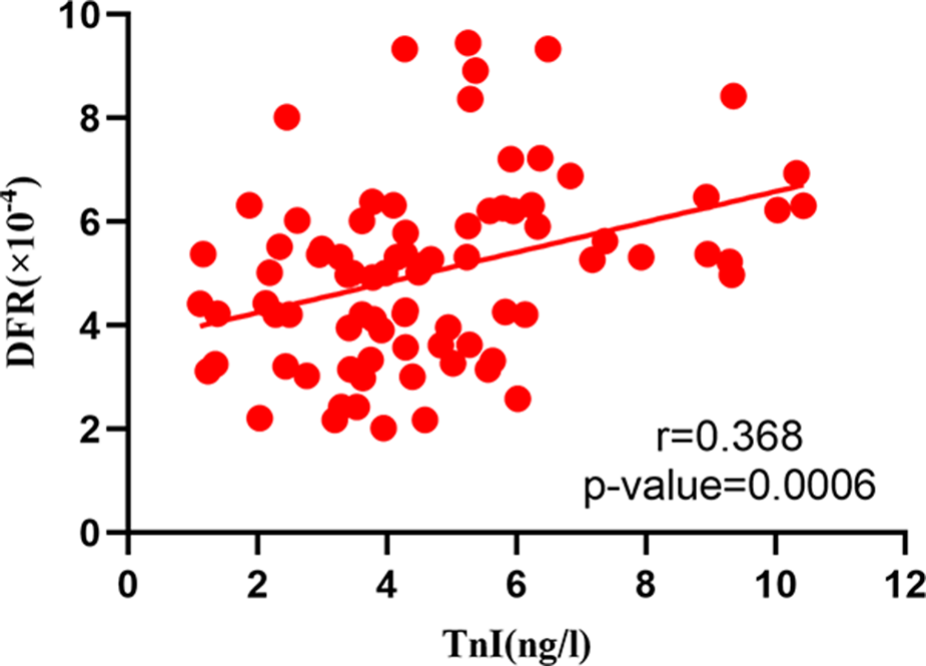
Correlation between serum TnI and DFR before operation.

**Figure 5:**
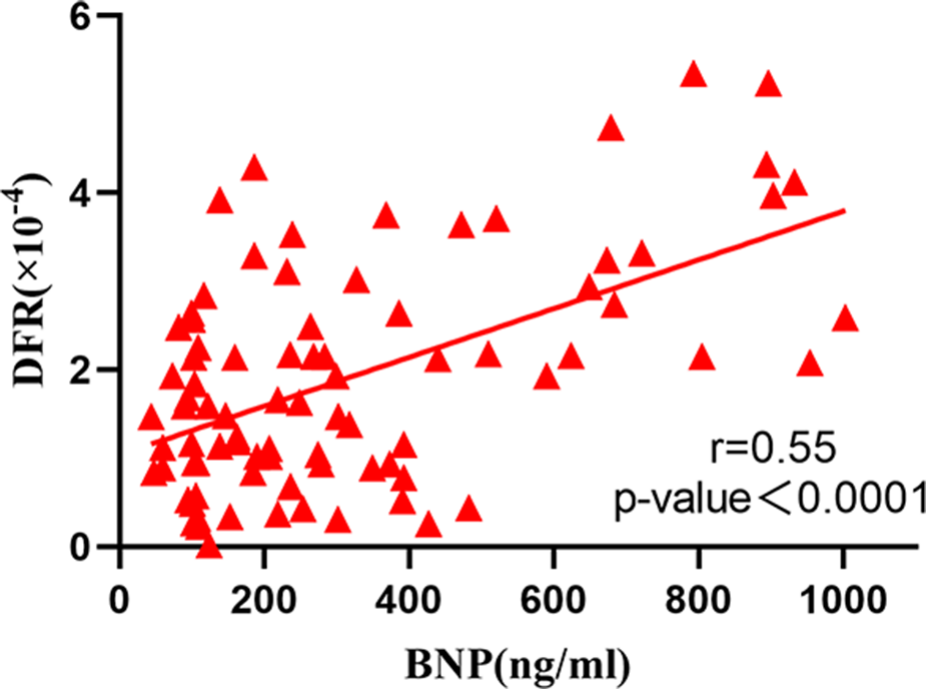
Correlation between serum BNP and DFR after operation.

**Figure 6:**
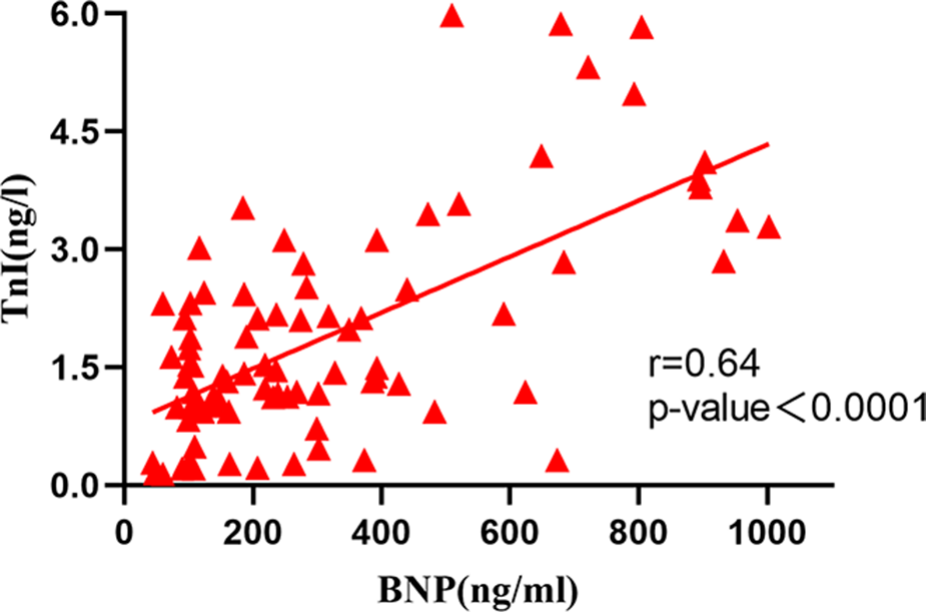
Correlation between serum BNP and TnI after operation.

**Figure 7:**
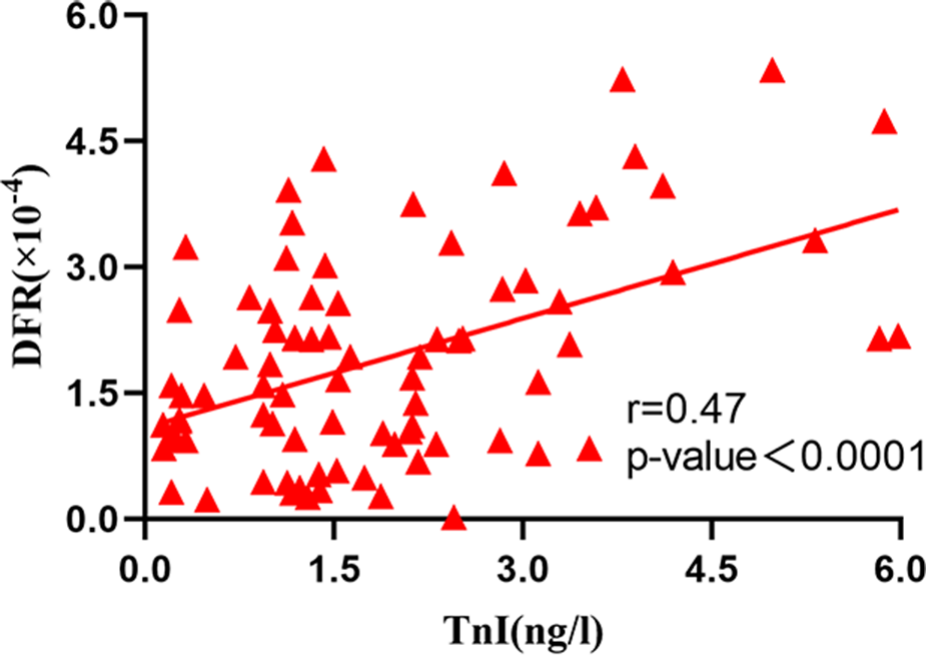
Correlation between serum TnI and DFR after operation.

The correlation analysis between serum BNP, TnI and DFR levels in 84 patients before and after surgery (Figures 2–7) showed that there was a positive correlation between BNP and TnI levels before and after surgery (*r* = 0.60, *P* < 0.001 vs. *r* = 0.55, *P* < 0.0001); There was a positive correlation between BNP and DFR before vs. after surgery (*r* = 0.61, *P* < 0.001 vs. *r* = 0.64, *P* < 0.0001); There was a positive correlation between TnI and DFR before vs. after surgery (*r* = 0.36, *P *< 0.0006 vs. *r* = 0.47, *P < *0.0001).

### Diagnostic value of serum BNP, TnI and DFR levels for APE

**Figure 8:**
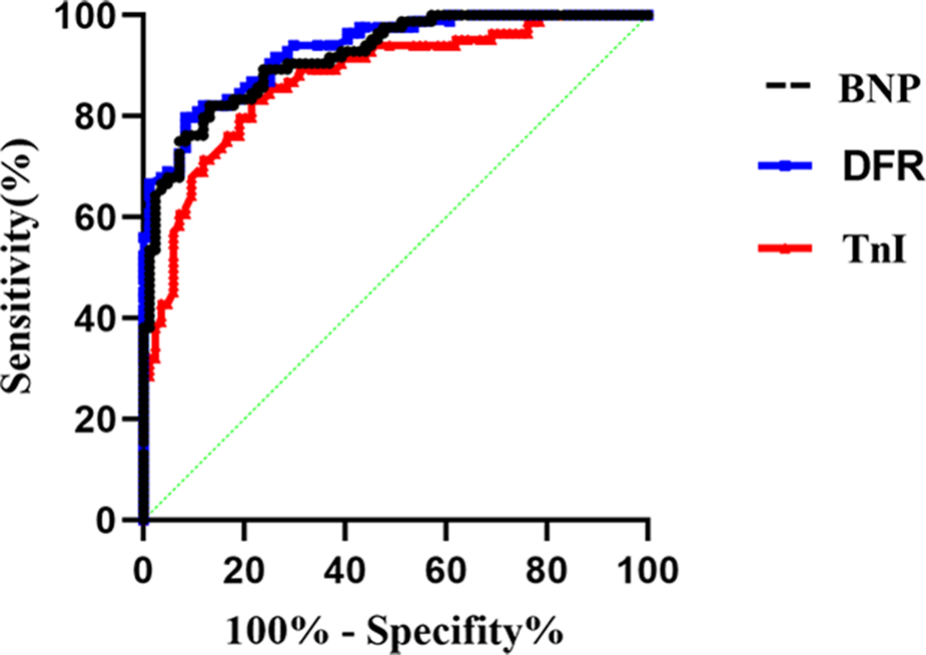
The ROC curve was used to analyze the serum BNP, TnI and DFR of APE patients.

The ROC curve was used to analyze the serum BNP, TnI and DFR of APE patients. The areas under the curves of BNP, TnI and DFR were 0.91, 0.87 and 0.93. The area under the curves showed that DFR was more effective in diagnosing BNP and TnI. The sensitivity and specificity of TnI were significantly higher than those of BNP and DFR. The difference was statistically significant (*P* < 0.001), as shown in Table 4 and Figure 8.

**Table 4:** Sensitivity, specificity and cut-off value of serum BNP and TnI and DFR levels.

Index	AUC	95% CI	*P*-value	Sensitivity%	Specificity%	Cut-Off Value%
BNP	0.91	0.878–0.957	<0.001	78.57	91.67	439
TnI	0.87	0.821–0.926	<0.001	91.67	95.71	2.96
DFR	0.93	0.895–0.965	<0.001	80.95	78.56	3.07

## Discussion

The severity of APE risk stratification is often assessed according to hemodynamics, resulting in different treatment methods and prognosis of APE. In recent years, with the improvement of laboratory biomarkers and imaging in the diagnosis of APE, the clinical misdiagnosis rate and missed diagnosis rate of APE have decreased significantly. The baseline data of this study is APE under different risk stratification after PMT and CDT treatment, and the patient's sex, age, trauma history and DVT history. The two groups of indicators have no statistical significance, excluding the influence of human factors.

BNP, TnI and DFR, as common indicators for diagnosis of thrombotic diseases (10), have the advantages of fast detection, low detection conditions and are not affected by the level of other indicators, and preliminary screening of cardio pulmonary vascular diseases. The serum BNP level of normal people is at a low level. When mechanical factors lead to the increase of right heart pressure, it will lead to the synthesis and release of BNP by ventricular myocytes, which is an effective indicator to evaluate the damage of right heart function (11). TnI has good sensitivity and specificity for myocardial damage assessment. When myocardial ischemia and hypoxia occur, the level of TnI will increase (12). In the high risk stratification of APE, when the volume of thrombus in the pulmonary artery is large, it will lead to a sharp increase in pulmonary artery pressure, increase the right heart load and right ventricular traction, and lead to cardiopulmonary circulation disorders, leading to a decrease in cardiac output and blood oxygen supply (13), leading to a significant increase in serum BNP and TnI levels. Some studies have shown that BNP and TnI can effectively assess the risk of APE and have a good prognosis (14). D-dimer is a degradation product of fibrin that increases in acute thromboembolic events, D-dimer concentrations can be used to diagnose or rule out PTE but its specificity is poor. Plasma fibrinogen is one of the most important factors in the coagulation cascade, such as haemodynamic impairment can Can cause it to rise. DFR can be used as an index of thrombogenic activity, and its data comes from the D-dimer/fibrinogen ratio. It can be used to evaluate the severity of thrombosis and stroke. When hypercoagulable state and secondary fibrinolytic activity increased in the body, the corresponding level of D-dimer increased significantly (15). FIB is the precursor of fibrin. In the final stage of coagulation, soluble fibrinogen can be converted into insoluble fibrin (16), so that blood can coagulate. Thrombosis can be diagnosed by detecting the plasma D-dimer and fibrinogen levels, calculating the ratio, and improving the diagnostic sensitivity (17). In this study, the serum BNP, TnI and DFR level in the high-risk stratification of APE was significantly higher than that in the Medium-risk group, the levels of serum BNP, TnI and DFR after PMT treatment of APE were significantly lower than those of CDT treatment. The main reason was that the pulmonary embolism was serious, leading to high pulmonary artery systolic pressure. The reduction of serum BNP, TnI and DFR levels in the medium and High-risk group s treated with PMT was significantly better than that of CDT, greatly reducing a series of complications caused by pulmonary hypertension caused by APE. At the same time, after PMT rapidly reduced the thrombus load in the main pulmonary artery, the blood perfusion in pulmonary artery increased, the oxygen content in systemic-pulmonary circulation increased, and the content of serum BNP released into pulmonary-peripheral arterial circulation decreased. After TnI reduced cardiomyocyte damage, it reduced right ventricular load and right ventricular traction, and increased right cardiac output. DFR reflects the balance of fibrinolysis/coagulation. When the secondary hyperfibrinolysis decreases, the level of DFR further decreases, which reduces the thrombus in pulmonary artery and branches, reduces pulmonary artery pressure and improves the prognosis.

According to the ICOPER registration study of 2,392 patients with pulmonary embolism (18), it was found that there was a contraindication of thrombolysis in patients with pulmonary embolism, so there was still a risk of sudden increase of pulmonary artery pressure and acute aggravation of cardiac load in patients with medium and high risk pulmonary embolism, and there was a risk of massive hemorrhage in systemic thrombolysis. This study discussed the advantages and disadvantages of PMT and CDT in the treatment of medium and high risk APE. It was mainly evaluated by the thrombus clearance rate, pulmonary artery pressure, average urokinase dosage, effective thrombolytic time and average hospital stay. The results showed that PMT's use of AngioJet vascular jet device for rapid thrombus clearance was significantly better than CDT. Through PMT's blood pumping, the thrombus load in the pulmonary artery was reduced, the local urokinase dosage was reduced, and the time required for treatment was also shortened, Shorten the whole treatment cycle of patients (19). Both PMT and CDT groups had chest tightness, decreased blood pressure, blood leakage around the vascular sheath and postoperative hemoglobinuria and other related complications during and after the operation. The incidence of complications in the two groups was similar, but the difference was not statistically significant. The main reason is that in PMT, AngioJet catheter was used to stimulate the blood vessel wall at high speed, resulting in vagus nerve excitation. During the operation, chest tightness, palpitation, blood pressure drop and other discomfort symptoms appeared, which were relieved automatically after the operation was suspended. As for hemoglobinuria after PMT, it is because the jet during AngioJet catheter operation has certain damage to red blood cells, and red blood cells are broken, resulting in hemoglobinuria. In CDT group, blood leakage and gross hematuria occurred at the puncture point. It was considered that the long-term use of urokinase and the amount of thrombolytic drugs were related, and the remission was achieved after reducing the amount of thrombolytic drugs. Some experts (20) believe that PMT and CDT can clear part or all of the thrombus, quickly open the blocked blood vessels, reduce the pulmonary artery pressure and right heart load, thereby reducing the mortality and recurrence rate. However, some patients have CDT contraindications, and the probability of massive hemorrhage caused by long-term catheter thrombolysis is as high as 20% (21). In this study, we followed up for half a year after PMT and CDT treatment, and found that the levels of serum BNP, TnI and DFR in the cure group were significantly lower than those in the effective group and the ineffective group, and the levels of serum BNP, TnI and DFR in the effective group were significantly lower than those in the ineffective group, indicating that both PMT and CDT can reduce the load of pulmonary artery trunk and branch thrombus, and reduce the right ventricular pressure. However, according to the changes of serum BNP, TnI and DFR levels, PMT is more effective than CDT in treating APE. The preoperative and postoperative biological indicators showed that the levels of serum BNP, TnI and DFR were positively correlated with each other, indicating that the serum of APE patients showed abnormally high expression and was related to the severity of the disease, which could reflect the prognosis and help clinicians to determine the patient's diagnosis, determine the condition and guide clinical treatment. At present, BNP, TnI and DFR are widely used in clinical screening and diagnosis of thrombotic diseases, but there is no report on the diagnostic efficacy of APE with high risk in APE. This study found that the area under the curve of BNP, TnI and DFR were 0.91, 0.87 and 0.93 respectively. When the best cut-off point of BNP > 438 ng/L was taken, the sensitivity and specificity of predicting death were 78.57 and 91.67, respectively;TnI cut-off point >2.96 ng/ml, the sensitivity and specificity of predicting death were 91.67 and 95.71 respectively. The sensitivity and specificity of DFR best cut-off point >3.07 in predicting death were 80.95 and 78.56, respectively. BNP, TnI and DFR have good sensitivity and specificity for predicting APE, but TnI and DFR have higher sensitivity and specificity for diagnosis. BNP, TnI and DFR have good clinical diagnostic value in the classification of APE risk stratification, and also provide a good prognostic method. This conclusion is consistent with the results of foreign clinical studies. It shows that when the serum levels of BNP, TnI and DFR are increased, especially when each index is greater than bestcut-offpoint, the risk of death increases significantly, which can be used as one of the indexes to judge the prognosis of patients in the early stage.

To sum up,the detection of serum BNP, TnI and DFR can reflect the risk stratification of different acute pulmonary embolism patients, which is helpful for later treatment and prognosis evaluation. PMT and CDT have satisfactory effects on the treatment of APE patients in the middle and high risk layers. Compared with CDT, PMT has the advantages of rapidly reducing the burden of pulmonary artery thrombosis, reducing the use of thrombolytic drugs, shortening the thrombolytic time, and reducing the incidence of pulmonary hypertension caused by APE. Its disadvantage is that it is easy to have vagal reflex, which cannot be effectively carried out in many hospitals in China. At the same time, if we can quickly detect BNP, TnI and DFR levels to determine the risk stratification of APE patients, it is helpful to differential diagnosis, treatment and prognosis evaluation. Because this study is a cooperation between two centers, the disadvantage is that the number of enrolled cases is limited. The diagnostic value of these indicators in APE severity grading needs to be verified by large-scale clinical trials and multi center studies.

## Data Availability

The datasets presented in this study can be found in online repositories. The names of the repository/repositories and accession number(s) can be found in the article/**Supplementary Material**.
